# Occult Ventricular Fibrillation Visualized by Echocardiogram During Cardiac Arrest: A Retrospective Observational Study From the Real-Time Evaluation and Assessment for Sonography—Outcomes Network (REASON)

**DOI:** 10.1016/j.acepjo.2024.100028

**Published:** 2025-01-13

**Authors:** Romolo Gaspari, Srikar Adhikari, Timothy Gleeson, Monica Kapoor, Robert Lindsay, Vicki Noble, Jason T. Nomura, Anthony Weekes, Dan Theodoro

**Affiliations:** 1 Department of Emergency Medicine, University of Massachusetts Medical School, Worcester, Massachusetts, USA; 2 Department of Emergency Medicine, University of Arizona, Tucson, Arizona, USA; 3 Department of Emergency Medicine, UH Cleveland Medical Center, Cleveland, Ohio, USA; 4 Department of Emergency Medicine, Christiana Care Health Systems, Newark, Delaware, USA; 5 Department of Emergency Medicine, Atrium Health - Carolinas, Charlotte, North Carolina, USA; 6 Department of Emergency Medicine, Washington University School of Medicine, St Louis, Missouri, USA

**Keywords:** cardiac arrest, echocardiography, ventricular fibrillation, point-of-care ultrasound, resuscitation

## Abstract

**Objectives:**

Cardiac arrest patients with a shockable rhythm are more likely to survive an out-of-hospital cardiac arrest (OHCA) compared with a nonshockable rhythm. An electrocardiogram (ECG) is the most common way to identify a shockable rhythm, but it can miss patients with clinically significant ventricular fibrillation (vfib). We sought to determine the percentage of nonshockable OHCA patients that demonstrated vfib on echo.

**Methods:**

Secondary analysis of echo images recorded from a prior study from our group, Real-Time Evaluation and Assessment for Sonography—Outcomes Network (REASON), a multicenter, observational study of OHCA patients presenting to the emergency department with nonshockable rhythms. Using ECG and echocardiogram images recorded during the initial cardiopulmonary resuscitation (CPR) pause, 2 independent emergency physicians determined the presence of vfib. Two experienced emergency physicians (R.G. and T.G.) reviewed echo images with adjudication by a third if necessary. ECG interpretation was unblinded to patient information. The primary outcome was the proportion of patients in occult vfib.

**Results:**

During the first CPR pause, reviewers noted occult vfib in 22/685 (3.2%; 95% CI, 2.1%-4.8%) subjects. Patients with ECG vfib (n = 55) were defibrillated immediately during the first pause in CPR, but no patients with occult vfib during the first pause in CPR were defibrillated. Subsequently, 50% (11 of 22) of occult vfib patients were defibrillated when ECG vfib was recognized during an ensuing pause in CPR.

**Conclusion:**

One in 33 OHCAs with a nonshockable ECG rhythm exhibits VF on echocardiogram. Patients presenting to the emergency department in a presumed nonshockable rhythm following OHCA may benefit from prompt defibrillation if personnel recognize occult vfib on echo.

**The Bottom Line**

A little over 3% of patients in cardiac arrest demonstrate ventricular fibrillation visible by echocardiogram, but is not visible by electrocardiogram. This occult ventricular fibrillation is being missed by relying on electrocardiogram alone.

## 1 Introduction

### 1.1 Background

Patients who present with a shockable rhythm are more likely to survive an out-of-hospital cardiac arrest (OHCA) than those with a nonshockable rhythm.^1^ Cardiac rhythm is almost uniformly determined using electrocardiography (ECG), but echocardiography (echo) can identify cardiac rhythm through direct visualization of myocardial activity.^2^^,^^3^

### 1.2 Importance

Rapid recognition of ventricular fibrillation (vfib) is important, as delays in defibrillation are associated with decreased survival.^4^, ^5^, ^6^, ^7^ Given the large difference in survival between vfib and other nonshockable rhythms, identification and defibrillation of occult vfib may decrease delays to defibrillation during OHCA, thereby impacting survival.

### 1.3 Goals of This Investigation

The objective of this current study was to describe the prevalence of occult vfib visualized on echo but not identified by ECG.

## 2 Methods

### 2.1 Study Design and Setting

This is a secondary analysis of data from the first Real-Time Evaluation and Assessment for Sonography—Outcomes Network (REASON) study.^2^ The first REASON study was a large prospective study that included patients from 2011 to 2014 who presented with nonshockable ECG rhythms after atraumatic OHCA across 20 sites in the United States and Canada (Clinical Trials Registry NCT01446471). The goal of the study was to determine the association between cardiac activity identified by echo and survival to hospital discharge. Patients underwent protocolized bedside echo. The study enrolled a total of 953 patients.

### 2.2 Selection of Participants

The parent REASON study included patients with nontraumatic OHCA who presented to the emergency department (ED) with a nonshockable rhythm and who underwent a minimum of 5 minutes of standard advanced cardiac life support (ACLS). The study protocol effectively excluded patients presenting with a shockable rhythm. For the current study, we included all patients in the first REASON study using available transthoracic echo images and accompanying interpretations of the ECG rhythm. We excluded patients without echo images, corrupted echo images, or images with poor quality.

### 2.3 Interventions

#### 2.3.1 Echo

In the first REASON study, echoes were performed and recorded at the initial pause in cardiopulmonary resuscitation (CPR) as well as subsequent pauses. CPR continued per ACLS protocol until halted by the clinical team for return of spontaneous circulation or perceived futility. Recorded transthoracic echo images were deidentified to remove all clinical information.

For the current study, we performed a blinded review of all echoes from the parent study. A retrospective echo image review was performed by 2 physicians: a physician with experience interpreting over 5000 cardiac arrest echoes, and a fellowship-trained physician with experience interpreting over 2000 cardiac arrest echoes. Physicians interpreted echo recordings as having cardiac activity, no cardiac activity, or vfib based on previously published definitions.^2^^,^^8^ Vfib on echo was defined as visible disorganized movement in the ventricular myocardium without a rhythmic cadence, sometimes described as an “earthquake” or “bag of worms” movement (see Video 1). Adjudication was performed in situations in which the initial interpretations were disagreed by a third fellowship-trained physician with experience interpreting over 300 cardiac arrest echoes.

#### 2.3.2 ECG

ECG findings were recorded as interpreted by emergency medical services personnel in the prehospital setting. ECG tracings obtained on patient arrival to the ED were also interpreted by unblinded clinicians involved in the resuscitation who had access to all patient information. ECG interpretation was recorded as asystole, pulseless electrical activity (PEA), vfib, ventricular tachycardia, paced rhythm, or perfusing rhythm with a pulse. As ECG tracings were not recorded, no review of ECG rhythm by research staff occurred retrospectively.

#### 2.3.3 Defibrillation

The patient was defibrillated per standard ACLS protocol according to the ECG rhythm strip but not the echo. As per protocol, defibrillation was performed using 360 joules on a monophasic defibrillator. As per protocol, defibrillation was performed using 360 joules on a monophasic defibrillator. CPR was restarted immediately in patients without an obvious change in rhythm with a palpable pulse.

### 2.4 Outcomes

The primary outcome was the proportion of occult vfib visualized by echo but not identified by ECG. Secondary outcome included survival to hospital discharge.

### 2.5 Data Analyses

Data were uploaded into a centralized database (REDCap). Agreement between 2 reviewers was measured using kappa analysis and excluded the adjudication reviewer. All statistical analyses were implemented with the use of JMP Pro software version 15 (SAS Institute Inc), with the exception of agreement, which was done with an online calculator (Graphpad.com).

## 3 Results

### 3.1 Characteristics of Study Subjects

**Figure 1:**
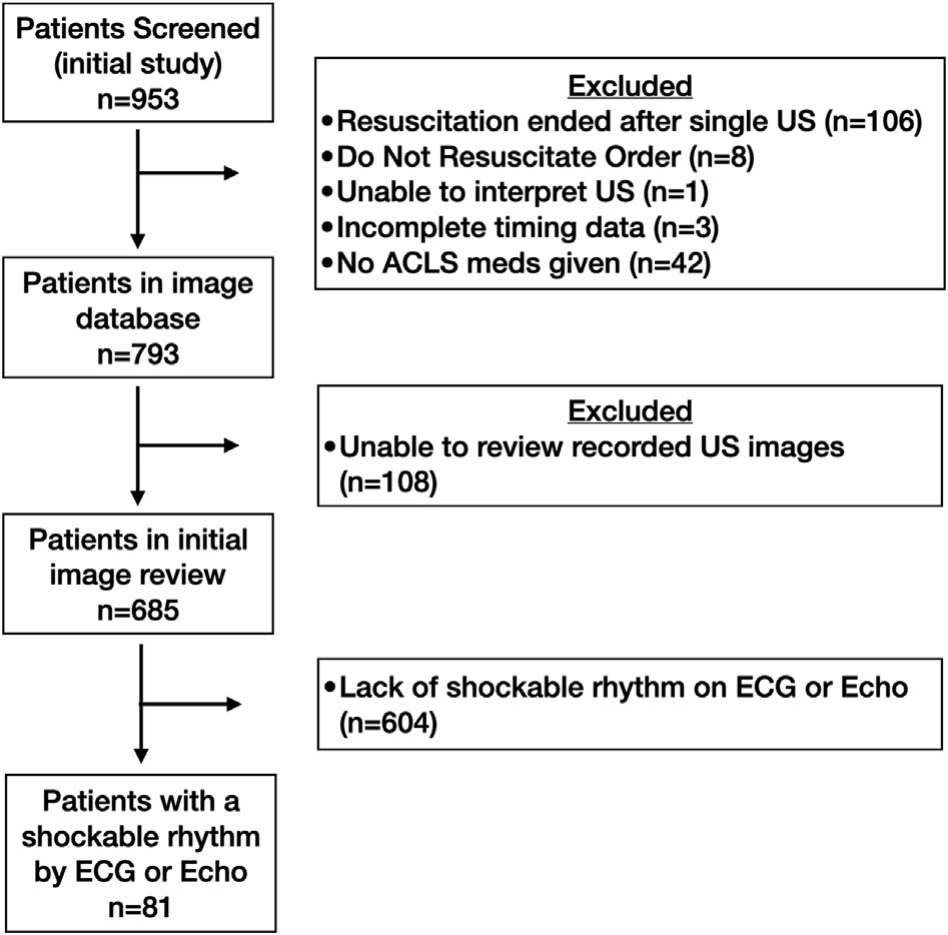
Study flow diagram. ACLS, advanced cardiac life support; ECG, electrocardiogram; Echo, echocardiogram; US, ultrasound.

A total of 953 patients arrived at the ED and were screened for inclusion in the initial study, and 685 patients were included in the current study (see Fig 1). Prior to ED arrival, prehospital personnel identified on-scene rhythms as asystole (45%), PEA (40%), vfib (14%), and sinus rhythm (1%). In the prehospital setting or during transport to the hospital, 24% of all patients included in this study were defibrillated by emergency medical services at some point prior to arrival to the ED. For patient characteristics, see the Table.

**Table:** Patient characteristics.

Characteristic of subjects	All patients (N = 685)
Demographics	
Age, mean (95% CI), y	64.5 (63.1-65.8)
Male, n (%)	419 (61.2)
Prehospital details of cardiac arrest, n (%)	
Bystander witnessed	334 (42.1)
Bystander CPR	185 (29.5)
Bystander AED	33 (4.8)
Time arrest to EMS arrival, mean (95% CI), min	8.8 (7.6-10.0)
In ED, details of cardiac arrest	
Length of resuscitation, mean (95% CI), min	25.1 (23.3-26.8)

AED, automated external defibrillator; CPR, cardiopulmonary resuscitation; ED, emergency department; EMS, emergency medical services.

### 3.2 Main Results

**Figure 2:**
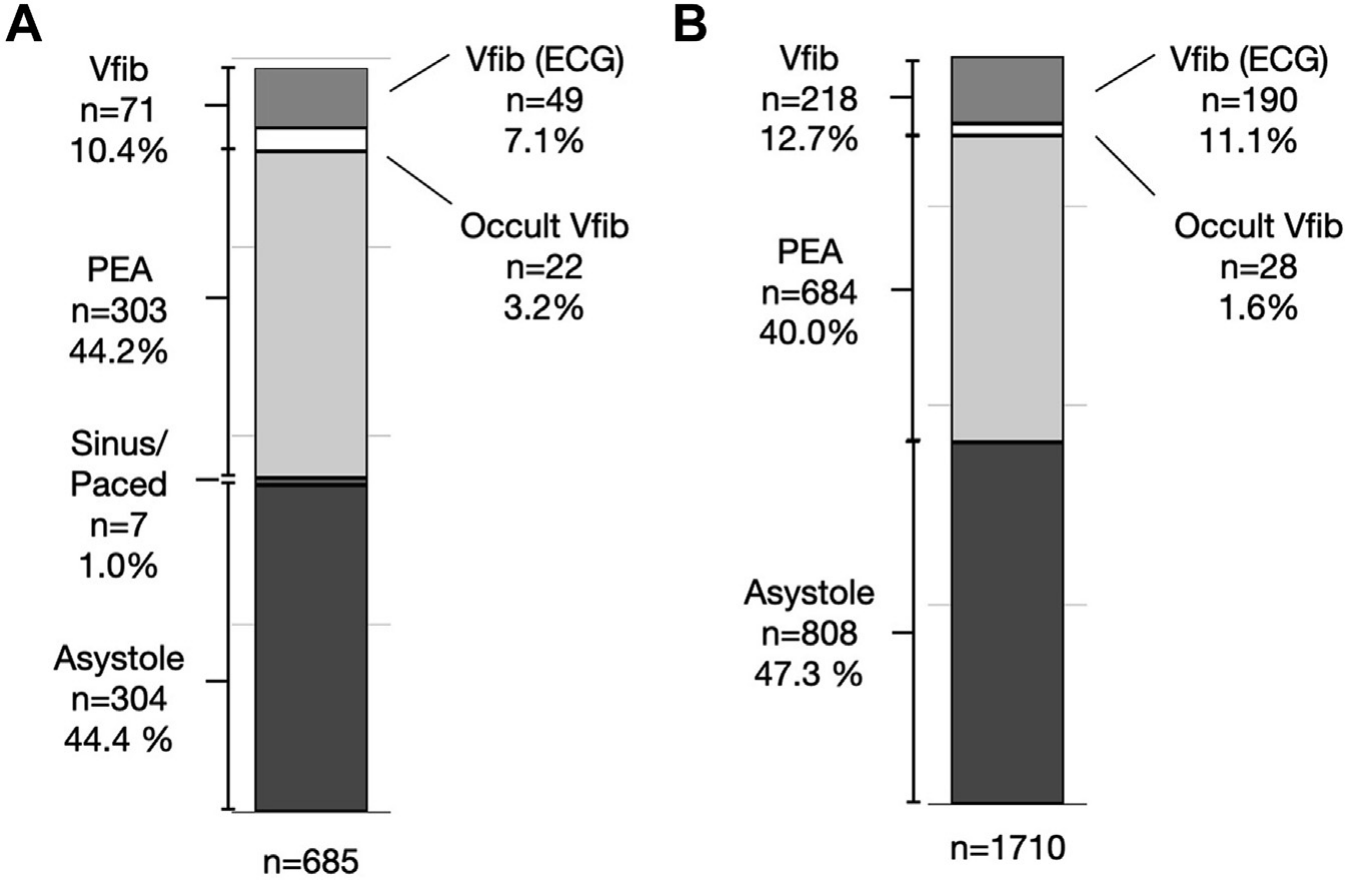
A. Cardiac rhythm for patients presenting with cardiac arrest. B. Cardiac rhythm for all pauses in cardiac arrest. ECG, electrocardiogram; Echo, echocardiogram; PEA, pulseless electrical activity; Vfib, ventricular fibrillation.

During the first pause in CPR, 71 of the 685 patients included in the study demonstrated either ECG vfib or occult vfib, with 3.2% (22 of 685) of vfib visible by echo but identified as a nonshockable ECG rhythm (occult vfib) (see Fig 2). Of the 22 patients with occult vfib, 11 demonstrated ECG asystole, and 11 demonstrated ECG PEA. None of the patients in occult vfib were defibrillated, but all patients with ECG vfib were defibrillated. Fifty percent (11 of 22) of patients with occult vfib during the initial pause were defibrillated during an ensuing pause when vfib was identified on ECG. Conversion to return of spontaneous circulation occurred in 4 of the 11 patients with occult vfib, who were eventually defibrillated (36.4%; 95% CI, 15%-64.8%). During all 1720 CPR pauses where echo and ECG rhythms were recorded, occult vfib occurred in 28 (1.6%) (see Fig 2).

Agreement between reviewers for all types of cardiac activity was excellent (kappa, 0.81; 95% CI, −0.77 to 0.85). Agreement between reviewers for the presence of vfib was moderate (kappa, 0.48; 95% CI, −0.35 to 0.62).

**Figure 3:**
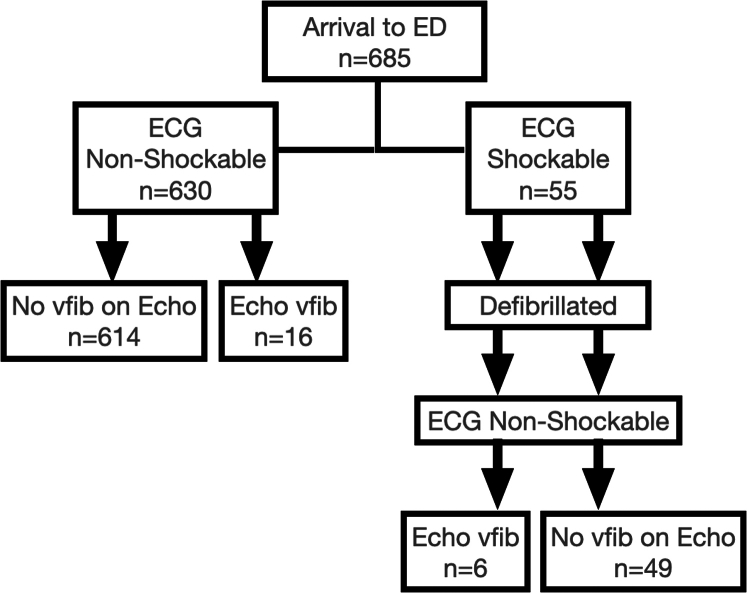
Cardiac rhythm and echocardiographic (Echo) activity during the first pause in cardiopulmonary resuscitation for patients presenting with cardiac arrest. ECG, electrocardiogram; ED, emergency department; Vfib, ventricular fibrillation.

Survival to hospital admission was not different when comparing patients with ECG vfib (18.2%) and occult vfib (18.8%). The highest rate of survival to hospital discharge was seen in occult vfib, but this is not statistically significant given the low number of patients (see Fig 3). Survival to hospital admission and hospital discharge for all patients with vfib were 18.3% and 1.4%, respectively.

## 4 Limitations

Our study has several limitations. We recorded ECG interpretations by clinicians but did not retrospectively review tracings for accuracy. Echo images were reviewed by expert sonographers with the ability to review recordings multiple times without a time constraint, and it is unclear if physicians managing an arrest would be able to identify all cases of occult vfib. However, echo recordings lasting 3 to 6 seconds represent only a fraction of the echo images visible to clinicians during the arrest who start recording after having identified the heart and already started image review before pressing record. Advances in imaging since the study took place may also impact the ability to identify occult vfib. Also, given the low number of events, we did not account for within-site or within-subject correlation, making the widths of our cis likely too narrow. Lastly, the initial study did not record neurologically intact survival. We recognize that this is the most important survival metric, but we only report survival to hospital discharge.

## 5 Discussion

Vfib masquerading as asystole or PEA has been noted previously,^9^^,^^10^ but the incidence of occult vfib during OHCA has not previously been described. Our findings support the idea that occult vfib is real, but additional research, including all OHCA patients, is required to provide a better estimate of the true prevalence. Our prevalence of ECG vfib in the study (7.1%) was lower than in prior publications; however, the initial study providing echo images for this manuscript excluded patients presenting to the ED with a shockable rhythm.^11^^,^^12^ Our population represents a different subset of individuals who arrived at the ED in PEA or asystole but converted to vfib between their arrival at the ED and the first pause in CPR.

It is important to note that 10.9% of patients defibrillated for vfib remained in occult vfib and were not subsequently defibrillated again until recognized later in the resuscitation. The authors considered the pauses with occult vfib linked to following pauses with ECG vfib. The delay in repeat defibrillation also likely contributed to the lower survival rate for vfib in this study (1.4%). Rapid defibrillation is a cornerstone of treating vfib and is responsible for the relatively larger survival rates for vfib in OHCA. The authors recommend that physicians who are comfortable identifying occult vfib should consider immediate defibrillation in patients with occult vfib. Future clinical studies exploring the survival benefit of defibrillating occult vfib would help direct resuscitation efforts.

At the time the patients were enrolled in the study, point-of-care ultrasound had a limited role in guiding resuscitation. It was common at that time for providers to use point-of-care ultrasound to identify a pericardial effusion for drainage or terminate resuscitative efforts (ie, stop CPR in patients without cardiac activity by echo). Some providers used echo to guide earlier initiation of intravenous adrenergic agents for occult hypotension,^8^ but this was not widespread. The authors are unaware of any clinicians who used echo to identify shockable rhythms at the time of the initial study. Given the moderate agreement for identifying vfib on echo, significant discrepancies in interpretation may limit the generalizability of our findings. Identifying occult vfib is relatively new for all clinicians, and attention during future education will be needed.

Any discussion on survival should be qualified by the recognition that vfib in this study represents a subset of the larger group of patients with vfib seen prehospital or in the ED. In this study, patients with occult vfib and ECG vfib demonstrated similar survival rates to hospital admission (18.8% and 18.4%, respectively). The survival rate to hospital admission for patients with occult vfib (18.8%) was higher than patients without both ECG vfib and occult vfib (13.2%) but with wide overlapping cis. This finding, though statistically weak, does raise the hypothesis that occult vfib and ECG vfib have similar prognoses and may respond similarly to immediate defibrillation. Further research is needed.

In conclusion, a percentage of vfib is unrecognized by ECG and subsequently not treated appropriately with defibrillation. Using echo to identify occult vfib has the potential to identify a patient population in OHCA that is currently being inadequately treated. Given the methodological limitations of this study, further research is needed to better quantify the prevalence and treatment options for occult vfib masquerading as a nonshockable rhythm.

## Author Contributions

R.G. conceived the study and designed the trial, as well as supervised the conduct of the trial and data collection. R.G., T.G., and M.K. were involved in echo interpretation. R.G. provided statistical analysis. R.G. drafted the manuscript, and all authors contributed substantially to its revision. R.G. takes responsibility for the paper as a whole.

## Funding and Support

This study was unfunded.

## Conflict of Interest

S.A. has book royalties from Springer and is a paid consultant for General Electric and EXO ultrasound. R.G. was a paid consultant to Butterfly Ultrasound Inc. The other authors have affirmed they have no conflicts of interest to declare.
