# Supplementary material for: Occult Ventricular Fibrillation Visualized by Echocardiogram During Cardiac Arrest: A Retrospective Observational Study From the Real-Time Evaluation and Assessment for Sonography—Outcomes Network (REASON)
Source: J Am Coll Emerg Physicians Open. 2025 Jan 13;6(1):100028. doi: 10.1016/j.acepjo.2024.100028 (PMC11853361; doi:10.1016/j.acepjo.2024.100028)
Supplement: Legend for Supplementary Video 1 [file mmc2.docx]

Supplementary Video 1 - Ventricular Fibrillation on Echocardiography - Echocardiographic images of the heart demonstrate a fibrillatory pattern of myocardial movement in both the right and left ventricle.
